# Dynamic Strain Modulation of a Nanowire Quantum Dot Compatible with a Thin-Film Lithium Niobate Photonic Platform

**DOI:** 10.1021/acsphotonics.3c00821

**Published:** 2023-09-28

**Authors:** Thomas Descamps, Tanguy Schetelat, Jun Gao, Philip J. Poole, Dan Dalacu, Ali W. Elshaari, Val Zwiller

**Affiliations:** † Department of Applied Physics, KTH Royal Institute of Technology, Roslagstullsbacken 21, 10691 Stockholm, Sweden; ‡ National Research Council of Canada, Ottawa, Ontario K1A 0R6, Canada; § Single Quantum BV, Rotterdamseweg 394, 2629HH Delft, The Netherlands

**Keywords:** quantum dots, nanowire, single photon source, surface acoustic waves, lithium niobate, integrated photonics

## Abstract

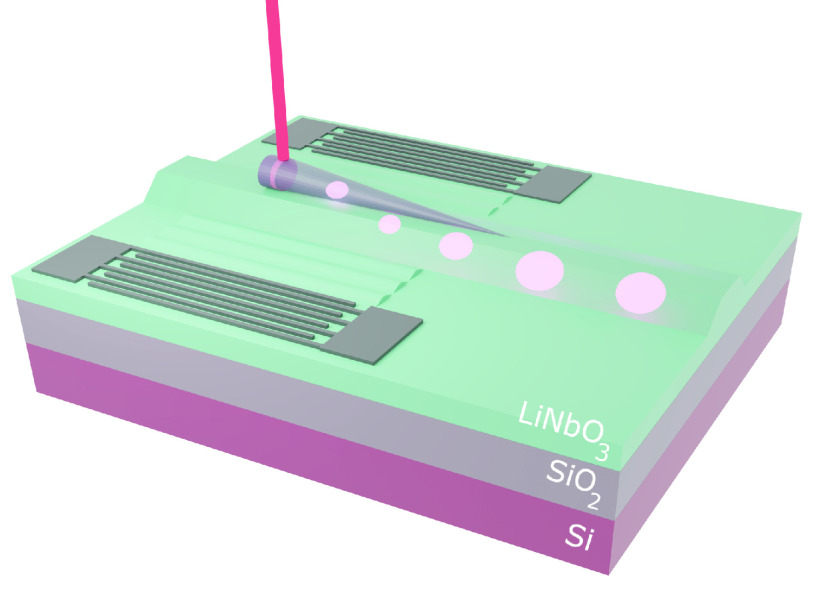

The integration of indistinguishable single photon sources in photonic circuits is a major prerequisite for on-chip quantum applications. Among the various high-quality sources, nanowire quantum dots can be efficiently coupled to optical waveguides because of their preferred emission direction along their growth direction. However, local tuning of the emission properties remains challenging. In this work, we transfer a nanowire quantum dot onto a bulk lithium niobate substrate and show that its emission can be dynamically tuned by acousto-optical coupling with surface acoustic waves. The purity of the single photon source is preserved during the strain modulation. We further demonstrate that the transduction is maintained even with a SiO_2_ encapsulation layer deposited on top of the nanowire acting as the cladding of a photonic circuit. Based on these experimental findings and numerical simulations, we introduce a device architecture consisting of a nanowire quantum dot efficiently coupled to a thin-film lithium niobate rib waveguide and strain-tunable by surface acoustic waves.

## Introduction

Quantum computation with on-chip photonic qubits has emerged as an important research area in quantum technologies.^1−4^ In addition to the manipulation and detection of individual photons, achieving deterministic integration of single and indistinguishable photon sources is essential for the scalability and complexity of quantum photonic circuits.^5−7^ Although more technologically challenging than monolithic approaches,^8−10^ the heterogeneous integration of high-quality quantum emitters offers the possibility of producing more efficient devices and incorporating additional functionalities that would be difficult to achieve in single material systems. Toward this direction, III/V semiconductor quantum dots (QDs),^11−15^ and defects in crystals^16,17^ or in 2D materials^18−20^ have been successfully transferred to Si, SiN, AlN, or LiNbO_3_ while maintaining good performances as single photon sources. In addition to their individual properties, multiple sources integrated on the same chip must be spectrally identical if indistinguishable photons are to be generated for linear quantum operations.^6^ Using a monolithic approach, two-photon interference between photons generated by two self-assembled QDs located in two different waveguides has recently been shown.^21^ Although surface scanning was successfully used to identify two QDs with naturally good spectral overlap, this method is unlikely to be efficient for QDs transferred to a host substrate. Indeed, even if the sources are carefully preselected on their original substrate, their spectral properties may change after integration due to a different charge and strain environment. Consequently, including an external tuning scheme for each source is essential to bring them into spectral resonance. Besides, finding naturally identical emitters is not a scalable approach, so having a tuning knob is desirable to relax the preselection step. Multiple tuning schemes have been explored based on temperature,^22^ magnetic field,^23^ electrical field via the quantum confined Stark effect,^24^ nanomechanical systems,^25^ strain field with piezoelectric materials,^26^ and surface acoustic waves (SAWs). The latter are generated from a piezoelectric material by an interdigital transducer (IDT) and can interact with a large variety of quantum systems. Coupling to superconducting qubits,^27,28^ coherent electron transport between gate-defined QDs^29^ as well as coherent acoustic control of optically active QDs^30,31^ have been demonstrated. In the latter case, the piezoelectric potential generated by the SAW can be used to transport photogenerated carriers to the QD,^32^ while the QD bandgap can also be modulated by the strain field of the SAW.^33−36^ Among the various transferable emitters or defects, InAsP QDs embedded in an InP nanowire (NW) with a taper-shaped InP shell stands out as a good candidate.^37,38^ Bright,^39^ high-purity,^40^ near transform-limited^41^ and indistinguishable single photons as well as entangled photons pairs^42,43^ have been preferentially emitted along the long axis of the NW with a Gaussian transverse mode. This emission profile is advantageous for efficient coupling to single-mode waveguides and fibers. Site-controlled growth with high yield^44^ is also particularly convenient for deterministic integration on photonic chips using pick-and-place techniques. On-chip routing, filtering, tuning, multiplexing, and detection of emitted single photons have been demonstrated on a SiN platform.^14,15,45−47^

In this work, we demonstrate the modulation of a single tapered nanowire QD with surface acoustic waves. The NW was transferred to a bulk LiNbO_3_ substrate and bonded to the substrate by van der Waals interactions. LiNbO_3_ offers a large electromechanical coupling coefficient (*k*^2^ ≈ 5%) making it particularly suited to modulate the emission of QDs at moderate RF power.^48^ We show that the emission wavelength of the quantum emitter can be tuned by more than 1 nm at the SAW resonance frequency, while the single photon purity is preserved. We then explore the influence of a SiO_2_ encapsulation layer on strain tuning performance. On the one side, this layer rigidly anchors the NW to the substrate, making it easier to manufacture a photonic chip if the NW is first transferred onto an unprocessed substrate. On the other side, once the NW is placed on the photonic waveguide, the SiO_2_ layer acts as a cladding layer. Based on these findings, we propose a device architecture where the NW is adiabatically coupled to a rib waveguide made from a LiNbO_3_ thin film and compatible with SAW-induced strain modulation. This fully integrated strain-tunable source will open the way for further on-chip manipulation and detection of photonic qubits.

## Methods

**Figure 1:**
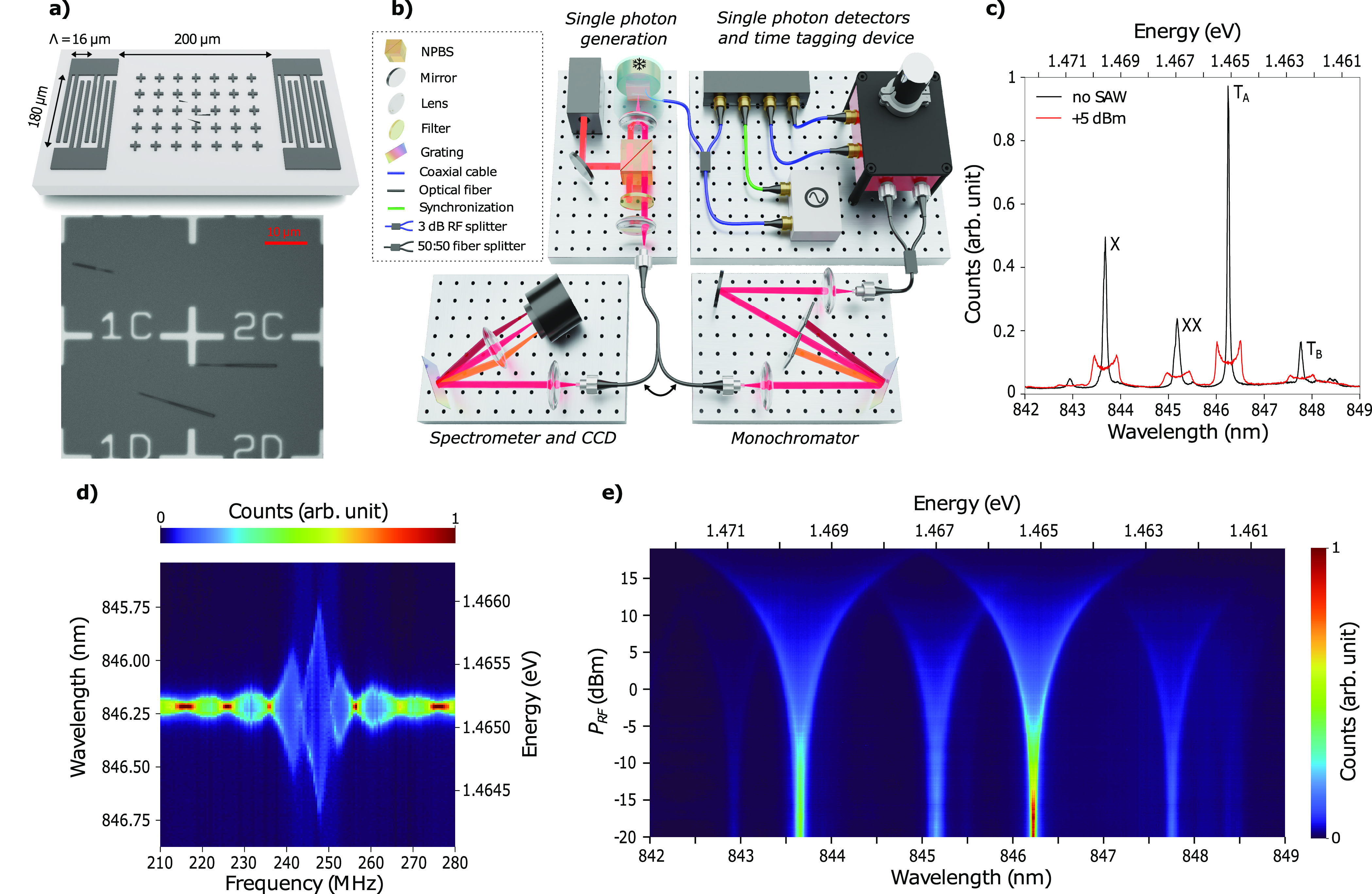
(a) Sketch of the surface acoustic wave delay line with an optical microscope image of individually transferred nanowires. (b) Optical setup for spectroscopy or time-resolved measurement (NPBS: nonpolarizing beam splitter). (c) Emission peaks of the nanowire QD without (black) and with (red) the SAW-induced modulation (*P*_RF_ = 5 dBm at 247.5 MHz). (d) Optical modulation of line *T*_*A*_ around the fundamental resonance frequency of the IDT (*P*_RF_ = 10 dBm). (e) Optical modulation as a function of *P*_RF_ (SAW generation at 247.5 MHz). For all measurements, the QD was continuously excited with a He–Ne laser at 150 nW.

The chip consists of two IDTs forming a 200 μm delay line patterned on a 128° Y-X cut LiNbO_3_ substrate by optical lithography and etching of a 60 nm-thick chromium layer (Figure 1(a)). The IDTs have a double-electrode structure to avoid internal reflections of the SAW.^49^ The electrodes are 2 μm wide, resulting in a spatial period Λ = 16 μm repeated 25 times per IDT. This configuration gives two SAW excitation frequencies, one fundamental resonance at *f*_1_ = *c*_LiNbO_3__/Λ = 249 MHz and a third harmonic at *f*_3_ = 3*c*_LiNbO_3__/Λ = 748 MHz, where *c*_*s*_ is the speed of sound in the substrate . The site-controlled InP NW embedding the InAsP QD was individually picked up with a micromanipulator and transferred^15^ onto the LiNbO_3_ chip within the delay line. The long axis of the NW is approximately parallel to the direction of propagation of the acoustic wave. In the following measurements, only the fundamental resonance is investigated. The sample was investigated at 1.8 K in a dry cryostat designed for confocal microphotoluminescence (PL) measurements and equipped with high-frequency cables (Figure 1(b)). A continuous wave He–Ne laser was focused with a microscope objective to excite the QD above band. The collected PL was dispersed by a 750 cm focal length spectrometer and detected by a liquid nitrogen-cooled charge-coupled device (CCD) camera. An analog signal generator was used to apply a sinusoidal radio frequency (RF) signal of adjustable power *P*_RF_ to one IDT of the delay line, while the other was floating. Figure 1(c) shows the PL spectrum of the QD without applying SAWs. From higher to lower energy, the four peaks are identified as the exciton *X*, the biexciton *XX*, and two trions, *T*_*A*_ and *T*_*B*_, based on power-dependent measurements (see Supporting InformationS1)^50^ and previous studies.^42^

## Results

When an RF signal is applied to the IDT, the radiated SAW couples to the QD through its strain field. Since the QD size is smaller by approximately 3 orders of magnitude than the wavelength Λ of the SAW, the strain field experienced by the QD can be considered uniformly distributed. The sinusoidal modulation of the strain field causes a modulation of the bandgap of the QD at the same frequency, resulting in the oscillation of the spectral lines around their unstrained energies. The time average of this oscillation gives a broad spectral line with peaks at the edges, as plotted in Figure 1(c) for a *P*_RF_ = 5 dBm sinusoidal signal at 247.5 MHz. For a given RF power, the tuning magnitude is largest when the SAW is generated close to the resonance frequency *f*_1_ of the IDT, as shown in Figure 1(d) for the *T*_*A*_ line. The main resonance peak is centered around 247.5 MHz, which corresponds to an effective speed of sound in LiNbO_3_ of . This main resonance spanning 20 MHz is split into three lobes separated by δ*f* = 6.6 MHz, since the delay line behaves as an acoustic cavity. The effective length of the cavity is hence  which is slightly larger than the physical length of the delay line due to the penetration of the SAW inside the two IDTs. A similar response is obtained for all of the other lines of the QD. At the SAW resonance frequency, detuning becomes observable for all of the peaks around *P*_RF_ = −15 dBm and can reach up to 1.5 nm at 15 dBm (Figure 1(e)). The broadening remains symmetric around the unstrained emission, indicating that high RF powers can be applied without inducing local heating of the QD. A good mechanical contact between the NW and the substrate is also maintained as the detuning does not drop even at high RF powers. However, the integrated line shape decreases for all peaks slightly above 5 dBm (see Supporting Information S2). The next experiments were carried out below this threshold.

**Figure 2:**
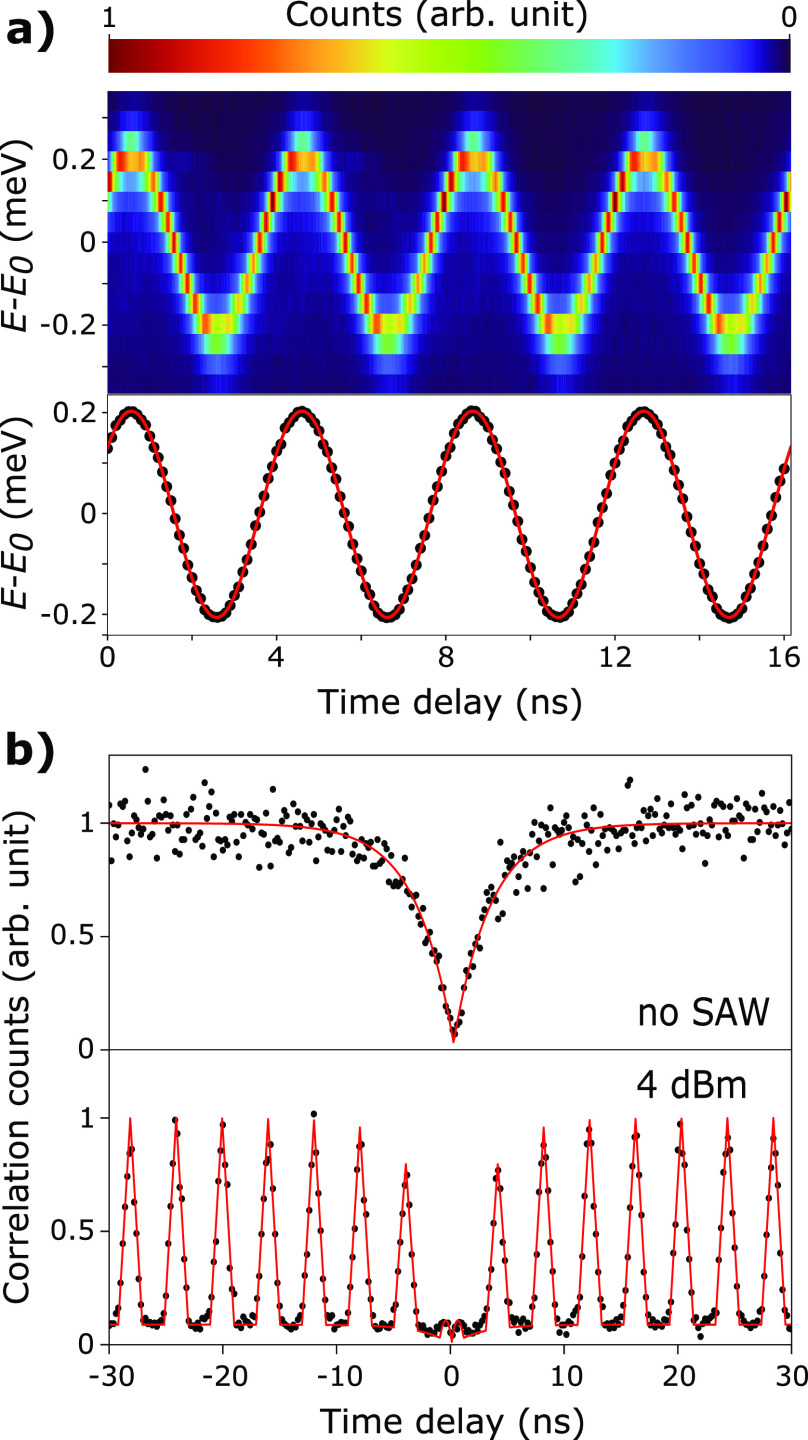
(a) PL spectra of the *T*_*A*_ line measured over 4 acoustic periods (top). The modulation with respect to the unstrained energy *E*_0_ was extracted and fitted by a sine function (bottom) with a period of 4.038 ns. (b) Second order correlation function of the *T*_*A*_ line without (top) and with (bottom) SAW interaction. In the latter case, the monochromator filtered at the energy corresponding to the maximum of the sine wave shown in (a). The fitting functions (red lines) are detailed in the main text. For all of the measurements, the QD was continuously excited with a He–Ne laser at 150 nW.

Time-resolved measurements were then performed to observe strain tuning over one acoustic cycle. As drawn in Figure 1(b), the PL signal was filtered by a monochromator (0.1 nm bandwidth) around the *T*_*A*_ emission line, detected by superconducting nanowire single photon detectors (time jitter around 20 ps), and counted by a time tagging device (time jitter below 10 ps) phase-locked to the signal generator. The sample was continuously excited with a He–Ne laser. A balanced power splitter was inserted at the output of the signal generator set at 247.5 MHz with an output power of 8 dBm. Half of the power triggered the counting module, while the other half drove the IDT. Over an acoustic cycle, pulses in the count rate can be observed as soon as the *T*_*A*_ emission wavelength falls within the filtering bandwidth of the monochromator. By sweeping the filtering window across the strain-tuned emission line, the time-dependent modulation can be reconstructed, as shown in Figure 2(a). This modulation is sinusoidal with a fitted frequency of 247.64 MHz, which closely matches the RF drive frequency. The absence of distortions in the sine wave shows that the tuning is only induced by the deformation potential, without contribution from the piezoelectric field via the quantum-confined Stark effect.^50^ The purity of the single photon source was then assessed by measuring the second-order correlation function of the *T*_*A*_ line in a Hanbury Brown-Twiss measurement (Figure 2(b)). The QD was continuously excited nonresonantly with a He–Ne laser. Without strain-tuning, *g*^(2)^(0) = 0.058 is measured experimentally, whereas a value of 0.0313 ± 0.019 is found by fitting the data to a biexponential decay function, hence demonstrating the high purity of the emitter. A SAW was then generated at *P*_RF_ = 4 dBm, while the PL was filtered at 846.58 nm, which corresponds to the wavelength where the modulation is maximum. Pulses were measured with a period of 4.039 ns and the peak at zero time delay was strongly suppressed. The experimental data were fit based on the detection probability model introduced by Gell et al.^51^ in which *g*^(2)^(τ) = *P*_*e*_(τ)*P*_*d*_(τ), where *P*_*e*_(τ) is the probability of the QD emitting a pair of photons with a temporal separation of τ (biexponential decay function), and *P*_*d*_(τ) is the pair-detection probability (convolution of two trains of top hat functions). The experimental point at zero delay *g*^(2)^(0) = 0.042 and the fitting parameter of 0.0114 ± 0.026 are very similar to the values obtained without modulation. This measurement under continuous wave excitation demonstrates the pulsed generation of high purity single photons over the tuning range induced by the strain modulation. A similar measurement was carried out when the monochromator filtered at 846.2 nm (Supporting Information S3) and shows that the period of the pulses is divided by two since the modulated emission falls within the monochromator bandpass twice over one acoustic cycle.

The ability to generate indistinguishable photons is also an important requirement for single photon sources and is typically measured in a Hong-Ou-Mandel two-photon interference experiment. Visibility as high as 83% has been demonstrated with the same nanowire QD system as used in this study.^52^ More recently, near Fourier transform-limited photons have been shown by carefully optimizing the growth conditions.^41^ For those two experiments, the nanowire QDs were standing up on the original surface and excited above band. Here, the nanowire QD studied was grown under the same optimized conditions, except for a 15 °C lower growth temperature of the core. After transferring the nanowire on the LiNbO_3_ substrate, the line width might broaden due to the proximity of the nanowire QD to a surface with a higher density of charges, degrading the degree of indistinguishability. Although not performed in this study, two-photon interference using the photons emitted by the strain-tuned nanowire could be carried out with the same spectral filtering as that implemented for the previously detailed time-resolved experiments. It is also compatible with resonant excitation schemes in order to reach high visibility by detuning the laser to the same wavelength as the filtering window,^33^ and by rejecting it at the monochromator output before the interferometer. Note that to avoid a potential change in polarization with the strain field, only one photon should be collected per acoustic cycle.

**Figure 3:**
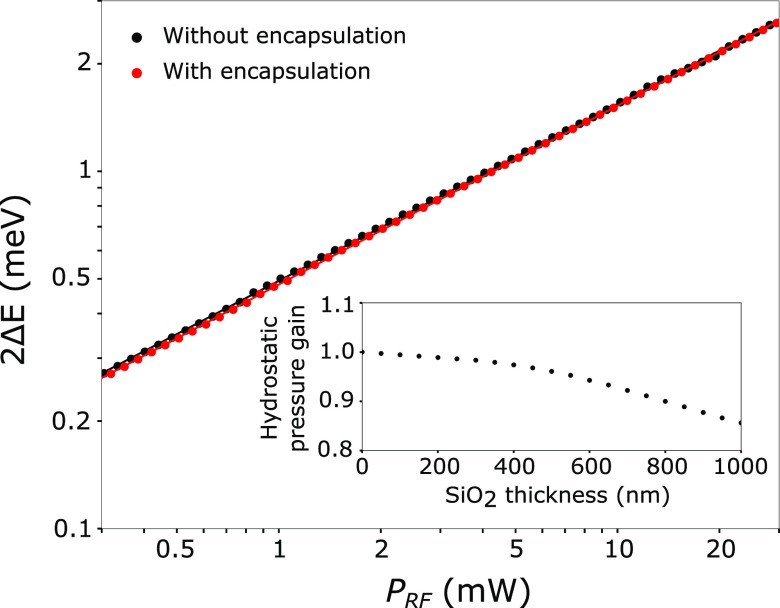
Strain-induced energy splitting of the *T*_*A*_ line as a function of *P*_RF_. The black and red points were taken before and after the deposition of SiO_2_ (320 nm) on top of the NW, respectively. The solid lines are linear fits. In both cases, the excitation power was 150 nW. The inset shows the evolution of the simulated hydrostatic pressure in the InP layer after the deposition of a SiO_2_ layer on top of the InP/LiNbO_3_ stack.

Next, the tuning of the QD emission was investigated after depositing a 320 nm-thick layer of SiO_2_ by PECVD on the chip. The oxide on top of the IDTs was locally removed to have the same resonance frequency and the same electrical to acoustic transduction as previously (see Supporting Information S4 for a comparison of the scattering parameters). This oxide layer bonds the NW to the substrate more rigidly than the van der Waals forces, which provides two benefits. On the one hand, it eases the fabrication of subsequent photonic structures, and on the other hand, it plays the role of a cladding layer when the NW is integrated with a lithium niobate on insulator (LNOI) waveguide. The impact of the SiO_2_ layer on the modulation was quantified by measuring the energy broadening as a function of RF power, similarly to Figure 1(e). The driving frequency chosen to maximize the acousto-optical coupling was still 247.5 MHz. Independent of the SAW power, the PL lines were blue-shifted by 1.92 nm, indicating a static strain introduced by the SiO_2_ layer onto the QD. Without the SAW, no significant broadening of the QD emission line could be resolved after encapsulation. The optomechanical modulation amplitude 2Δ*E* was extracted from the data by fitting to a time-integrated oscillating Lorentzian emission line.^53^ The optomechanical response 2Δ*E* is plotted as a function of the driving RF power in logarithmic scale before and after SiO_2_ encapsulation (Figure 3). In both cases, the strain-induced broadening follows the power law , where α = 0.5001 ± 0.0003 with SiO_2_ and α = 0.4951 ± 0.0004 without. These two coefficients are very close to the ideal value α = 0.5 for a deformation potential coupling,^54^ demonstrating that the observed broadening is essentially due to optomechanical coupling. For a given RF power within the range −5 to 15 dBm, the encapsulation resulted on average in a broadening smaller by around 1.8%. The influence of the encapsulating layer thickness on the acousto-optical coupling was studied based on 2D FEM frequency domain simulations with COMSOL. The SAW was excited at the fundamental resonance of a bulk 128° Y–X cut LiNbO_3_ substrate, and propagated along the *X*-axis toward a NW placed on top of the substrate. The NW is modeled as a linear elastic slab of InP with a thickness of 200 nm, corresponding to the diameter of the NW at the location of the quantum dot. A linear elastic SiO_2_ layer of variable thickness was added to top of this slab. The SAW-induced hydrostatic pressure *p* defined as *p* = −*E*_*Y*_ε, where ε and *E*_*Y*_ are the trace of the strain tensor and the Young’s modulus respectively, was computed at the center of the InP layer.^55^ The density of the PECVD oxide was set to 2200 kg/m^3^, its Poisson’s ratio to 0.24^56,57^ and the Young’s modulus was considered a fitting parameter to match our model to the experimental data. A Young’s modulus of 82 GPa was obtained for our film. The influence of the SiO_2_ thickness on the hydrostatic pressure inside the InP layer was then simulated and is shown in the inset of Figure 3. For thicker encapsulation layers, the hydrostatic pressure decreases in the InP layer as the SAW is no longer localized at the top of the bulk LiNbO_3_ but also propagates at the surface of the SiO_2_ (see Supporting Information S5 for strain profiles).

## Discussion

**Figure 4:**
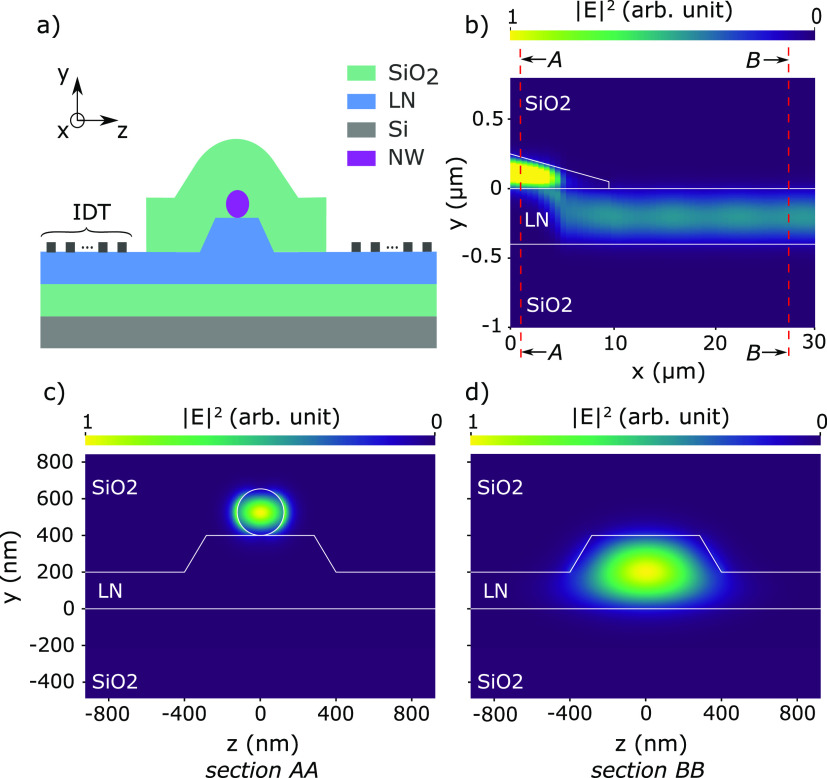
(a) Sketch of the nanowire QD placed on top of a thin-film LiNbO_3_ rib waveguide with IDTs on each side for acousto-optical modulation. The SiO_2_ layer acting as a cladding and encapsulation layer for the nanowire is locally removed in the IDT region. (b) Optical TE mode transfer between the tapered NW and the rib waveguide. (c) Cross section AA showing the confined optical mode in the NW before the mode transfer region. (d) Cross section BB representing the optical mode confined in the rib waveguide after the mode transfer region. The refractive indices at 850 nm are *n*_LiNbO_3__(e) = 2.17, *n*_LiNbO_3__(o) = 2.25, *n*_SiO2_ = 1.45, and *n*_InP_ = 3.46. The axes correspond to those of a Y-cut LNOI.

Guiding the light emitted by the SAW-modulated QD would enable on-chip single photon manipulation and detection. Toward this direction, we propose a novel architecture shown in Figure 4(a). The tapered NW is placed on top of an X-oriented rib waveguide made from a 400 nm-thick Y-cut LNOI wafer. The NW is modeled as a 10 μm-long truncated cone with a base diameter of 200 nm and a top diameter of 50 nm. The rib waveguide has a height of 200 nm, leaving a base of 200 nm to drive SAWs. The thick buried oxide (3 μm) efficiently confines the optical mode in the rib waveguide, while the NW is encapsulated by a 320 nm thick conformal SiO_2_ layer. The tapered shape of the NW favors an adiabatic mode transfer of the TE and TM modes of the NW^15^ to the fundamental TE and TM modes of the waveguide, respectively (Figure 4(b–d)). Finite-difference time-domain simulations (Lumerical) were conducted to calculate the coupling efficiency of the device assuming lossless materials. For a 800 nm-wide rib waveguide, a coupling efficiency of 95.1% for the TE modes and 98% for the TM modes was obtained. This large waveguide geometry further facilitates the placement of the NW by using a manipulator. The efficiency of other waveguide geometries can be found in Supporting Information S6. The IDTs excite SAWs along the *Z*-axis of the crystal, which has the largest electromechanical coupling coefficient. Unlike in the experimental section, the NW is now perpendicular to the propagation direction of the SAW, but this change in orientation does not significantly affect the strain-induced coupling as shown in Supporting Information S7.

**Figure 5:**
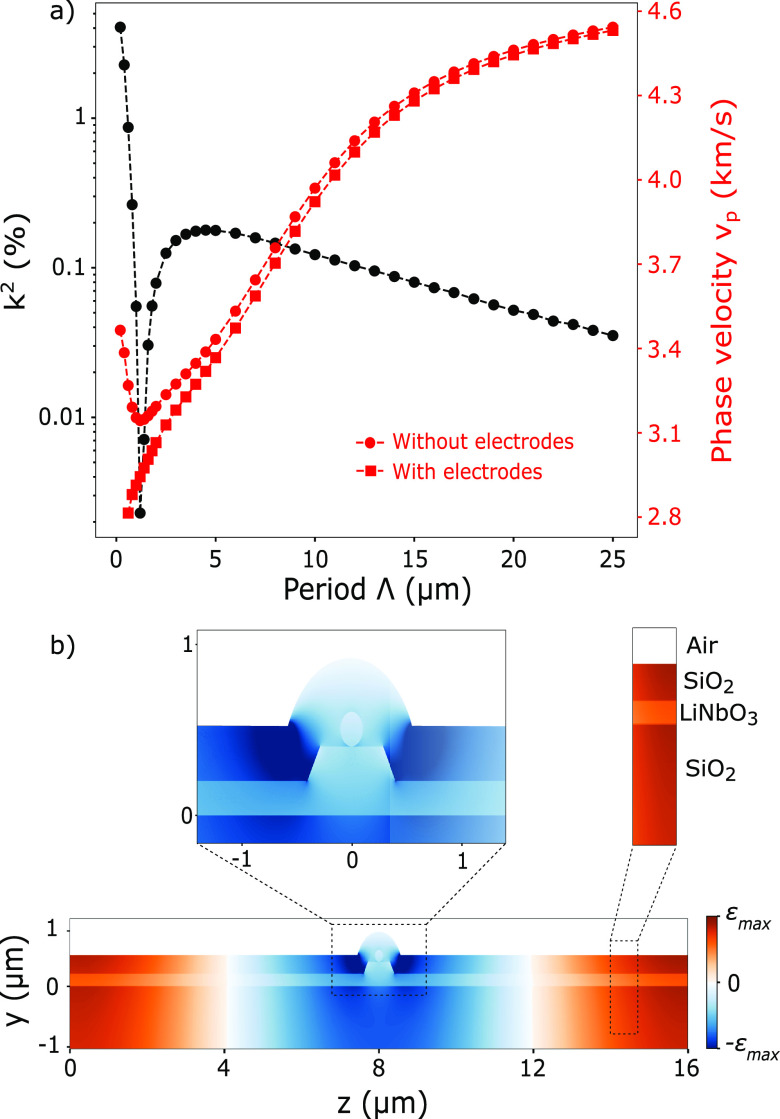
(a) Influence of the IDT period Λ on the electromechanical coefficient *k*^2^ (black) and the Rayleigh wave phase velocity *v*_*p*_ (red) for Y–Z LNOI (200 nm LiNbO_3_ – 3 μm burried SiO_2_) without SiO_2_ encapsulation. (b) Strain profile generated by a SAW with a period of 16 μm. The NW is placed on top of a waveguide with the same geometry as in Figure 4 and is encapsulated by 320 nm of SiO_2_. The simulation axes correspond to those of the crystal.

The properties of the surface acoustic wave depend on their propagation medium. For the LNOI under consideration, the Rayleigh wave phase velocity *v*_*p*_ and the electromechanical coupling coefficient *k*^2^ are now determined by the thicknesses of the LiNbO_3_ and burried SiO_2_ layers. These two parameters were assessed by 2D FEM simulations with COMSOL in eigenmode outside the waveguide (the encapsulating SiO_2_ layer is not considered in this simulation, since it is present only in the waveguide region). Two electrodes (60 nm of chromium) are placed on the surface of the modeled LiNbO_3_/SiO_2_/Si layer stack. By grounding one electrode while setting the other to a floating potential, two propagating modes, one symmetric and one antisymmetric, are identified. The Rayleigh wave phase velocity is derived as *v*_*p*_ = (*f*_*S*_ + *f*_*A*_)·Λ/2, where *f*_*S*_ and *f*_*A*_ are the frequencies of the symmetric and antisymmetric modes, respectively. The electromechanical coupling coefficient is computed as *k*^2^ = 2(*v*_*p*,*o*_ – *v*_*p*,*s*_)/*v*_*p*,*o*_, where *v*_*p*,*o*_ and *v*_*p*,*s*_ are the phase velocities when the surface is open and shorted, respectively.^58^ For 200 nm LiNbO_3_ and 3 μm buried SiO_2_, the influence of the IDT period is shown in Figure 5(a). For a period comparable to the LiNbO_3_ thickness, the coefficient *k*^2^ converges to the bulk value around 4.1%. Similar optomechanical coupling as demonstrated above is therefore expected. The phase velocity converges to the phase velocity of bulk LiNbO_3_ around 3490 m s^–1^ when no physical electrodes are on the surface. Adding the electrodes slows down the wave drastically due to their weight, and the modes are distorted for very short periods. Up to Λ < 1.5 μm, the wave propagates in the LiNbO_3_ film but partially leaks into the SiO_2_ layer, resulting in a drastic decline of the coefficient *k*^2^ and slower wave velocities. For Λ > 1.5 μm, the phase velocity increases as the wave penetrates deeper into the SiO_2_ to reach the Si substrate which is a faster medium than SiO_2_ and LiNbO_3_. The coefficient *k*^2^ also increases to a maximum value of 0.17% due to the waveguiding effect in the LiNbO_3_ and SiO_2_ layers, which couples more vibration in these layers.^59^ For Λ > 5 μm, this confinement effect declines as the wave propagates deeper in the silicon, resulting in a *k*^2^ drop. For a period of 16 μm, *v*_*p*_ = 4350 m s^–1^ and *k*^2^ = 0.074%. This value of *k*^2^, although smaller than the bulk scenario, is comparable to that of bulk GaAs (≈ 0.07%) on which modulation of In(Ga)As QDs has been demonstrated.^60^ Then, we evaluate the impact of the waveguide and encapsulation oxide (320 nm) on the coupling between the SAW and the NW by computing the strain at the center of the NW (Figure 5(b)). Its amplitude drops by 43% when the NW is placed on top of the waveguide compared to the case when it is on the surface of a 200 nm thick LNOI (Supporting Information S7). This loss mostly originates from the dome of SiO_2_ around the waveguide, which creates a local strain minimum. Furthermore, we estimate from the simulations that the strain at the center of the encapsulated NW on top of the LNOI waveguide will be 24 fold smaller than the strain in an encapsulated nanowire placed on the surface of the bulk LiNbO_3_. This 28 dB loss translates to a spectral shift of 0.12 nm at a 19 dBm RF power according to Figure 1(e). Therefore, more RF power will be necessary to shift the emission of the NW on LNOI waveguide to partially compensate for this loss. This has the detrimental effect of increasing the heat load on the sample, which can nonetheless be mitigated by RF pulsed excitation (see Supporting Information S2 for further discussion about operation at high RF power). Besides increasing the RF power, the modulation efficiency can be improved in several ways. First, the SAW frequency can be increased, as it allows to reach a larger electromechanical coupling—at Λ = 5 μm, *k*^2^ = 0.18%, more than twice the value at 16 μm—and generates a stronger strain since strain scales linearly with the SAW frequency. Second, the geometry of the IDT can be optimized to launch the SAW only in the direction of the QD instead of the bidirectional emission of the design considered in this study.^61^ Thus, a loss of 3 dB could ideally be avoided. Another option would be to use a focusing IDT and place the QD at the acoustic waist in order to strengthen the acousto-optic interaction.^30^ Third, the local strain minimum in the SiO_2_ dome can be mitigated with a thicker encapsulation layer (see Supporting Information S7).

Other strain-tuning techniques have been reported to shift the QD emission in integrated photonic circuits. Elshaari et al.^45^ showed a 0.8 nm spectral shift of a nanowire QD emission integrated in a photonic circuit fabricated on top of a PMN–PT substrate. Large voltages (600 V) were applied to observe this shift, but the power consumption remained low. The strain was equally applied to the source and all circuitry elements, hence, limiting selective tuning of individual components. This issue could be overcome by working with thin PMN–PT films,^62^ but their integration with photonic circuitry will likely involve a sophisticated fabrication process and, to the best of our knowledge, has yet to be demonstrated. Capacitive micromechanical systems^63^ have also been used to tune the emission of QDs in a III/V platform^64^ and color centers.^16,65^ Reasonably low voltages had to be applied to observe a spectral shift, while the power consumption also remained low since it mainly resulted from leakage currents. Nevertheless, this approach relies on fragile suspended membranes and can suffer from a substantial hysteresis.^66^ The tuning scheme presented here will comparatively require a higher power consumption, but IDTs can easily be fabricated with a high success rate near each emitter to tune them independently.

## Conclusion

In conclusion, we demonstrated the dynamic modulation of the emission of a quantum dot nanowire transferred to a LiNbO_3_ substrate using surface acoustic waves. The strong optomechanical coupling results in a wide optical tuning range at moderate RF powers, thereby avoiding heating of the sample, charge transport, or Stark effect modulation. The purity of the single photon source was preserved during the dynamic modulation process. Adding a SiO_2_ encapsulation layer on top of the nanowire does not significantly deteriorate the modulation and presents two benefits. It firmly anchors the nanowire to the substrate and acts as a cladding layer when the nanowire is integrated on a waveguide. Toward this idea, we proposed an architecture to adiabatically transfer the light emitted by the quantum dot into a rib waveguide made from LNOI compatible with strain modulation. The use of a LiNbO_3_ thin film combined with an encapsulated waveguide reduces the acousto-optical coupling to a level of performance comparable to that of bulk GaAs for a SAW period of 16 μm. Operating at a higher SAW frequency, a better IDT design, and a reasonably thicker SiO_2_ encapsulation layer are identified as solutions to improve the modulation efficiency. The scheme presented can be extended to other types of nanowire quantum dots, such as GaAs QD in AlGaAs nanowire emitting close to 780 nm which can be strain-tuned to match the D2 cycling transition of ^87^Rb.^67^ More generally, this approach is compatible with other emitters like semiconductor quantum dots in a tapered nanobeam or defects in 2D materials and crystals. In addition to the source modulation, the properties of other photonic components such as Mach–Zehnder interferometers and ring resonators can also be acoustically modulated to engineer reconfigurable quantum photonic circuits on LNOI.

## Supporting Information Available

Power-resolved PL; Spectral modulation at strong microwave power; Additional Hanbury-Brown Twiss measurement at *P*_RF_ = 4 dBm; Insertion loss before and after SiO_2_ deposition; Strain profile for a thick encapsulation layer; Simulation of the nanowire-waveguide optical coupling; Additional strain profiles for the LNOI architecture; Line width of the *T*_*A*_ peak before and after SiO_2_ deposition; Lifetime measurement of *T*_*A*_; Strain modulation at 735 MHz; Estimation of the maximum number of single emitters tuned with SAWs on the same chip (PDF)

## Notes

A preprint version of this work is available: Descamps, T.; Schetelat, T.; Gao, J.; Poole, P. J.; Dalacu, D.; Elshaari, A. W.; Zwiller, V. Dynamic strain modulation of a nanowire quantum dot compatible with a thin-film lithium niobate photonic platform. arXiv, June 9, 2023, 2306.05798. DOI: 10.48550/arXiv.2306.05798 (accessed on September 14, 2023).
