# Supplementary material for: Dynamic Strain Modulation of a Nanowire Quantum Dot Compatible with a Thin-Film Lithium Niobate Photonic Platform
Source: ACS Photonics. 2023 Sep 28;10(10):3691–9. doi: 10.1021/acsphotonics.3c00821 (PMC10588554; doi:10.1021/acsphotonics.3c00821)
Supplement: Supplementary file 1 — ph3c00821_si_001.pdf [file ph3c00821_si_001.pdf]

Supporting Information

Dynamic strain modulation of a nanowire quantum dot compatible with a thin-film lithium niobate photonic platform

Thomas Descamps[1,a], Tanguy Schetelat[1], Jun Gao[1], Philip J. Poole[2], Dan Dalacu[2], Ali W. Elshaari[1], Val Zwiller[1,3,b]

[1]Department of Applied Physics, KTH Royal Institute of Technology, Roslagstullsbacken 21, 10691 Stockholm, Sweden
[2]National Research Council of Canada, Ottawa, Ontario K1A 0R6, Canada
[3] Single Quantum BV, Rotterdamseweg 394, 2629HH Delft, The Netherlands

mail: [a]descamps@kth.se; [b]zwiller@kth.se

This document includes: pages 1-9, figs. S1-S11.

Contents:

1. **Power-resolved PL**
2. **Spectral modulation at strong microwave power**
3. **Additional Hanbury-Brown Twiss measurement at P_{RF} = 4 dBm**
4. **Insertion loss before and after SiO_2 deposition**
5. **Strain profile for a thick encapsulation layer**
6. **Simulations of the nanowire-waveguide optical coupling**
7. **Additional strain profiles for the LNOI architecture**
8. **Linewidth of the peak T_A before and after SiO_2 deposition**
9. **Lifetime measurement of T_A**
10. **Strain modulation at 735 MHz**
11. **Estimation of the maximum number of single emitters tuned with SAW on the same chip**

1. Power resolved PL

In Fig. S1, the integrated count is shown as a function of the laser excitation power. The blue, green, orange and red datasets correspond to the lines labeled X, XX, T_A and T_B in the main text, respectively. X and T_A exhibit the lowest slopes, while XX and T_B have the largest ones [1].

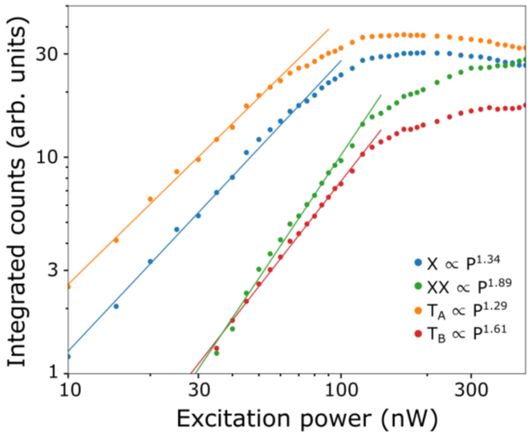

Fig. S1: Power dependent measurement of the 4 emission lines identified in the nanowire quantum dot excited with a HeNe laser. The points are experimental values and the solid lines linear fits.

2. Spectral modulation at strong microwave power

The trend of Fig.1e) in the main text does not show saturation or a decrease of the tuning amplitude range at the maximum RF power that we applied, which indicates that the spectral shift can be further increased with larger strain fields. However, there is an upper bound to the RF power that can be applied. If we only consider the behavior of the IDT, degradation of the electrodes occurs at large RF power by acoustomigration of the metal grains [2,3], which shifts the resonance frequency over time. At even larger power, the IDT is completely damaged by electrical breakdown. The power level required to initiate these two effects depends on the metal thickness and electrode geometry, but if we consider that the damage is irreversible around 30 dBm [3], this would mean a maximal shift of 11 nm. However, other factors are probably going to further limit the maximum RF power. First, large RF power is going to heat the QD which will manifest as a redshift of the emission lines [4]. For SAWs applied on a GaAs substrate, Buhler *et al.* estimated that 23 dBm continuous RF power would lead to a sample temperature around 55 K. This will result in a drop of performance regarding the single photon source (visibility of the two-photon interferences). This effect can nonetheless be mitigated with pulsed RF drive. Second, we noticed that the integrated count rate starts to drop above 5 dBm as shown in Fig. S2. A trade-off between the spectral shift and the count rate may have to be made for some applications.

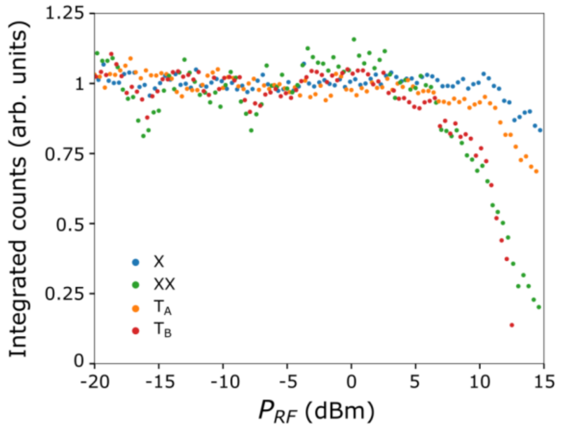

Fig. S2: Integrated count rates of the four peaks represented in Fig. 1(e) in the main text. Each peak was fitted by a time-integrated oscillating Lorentzian. The intensity starts to drop for all the peaks slightly above P_{RF} = 5 dBm.

3. Additional Hanbury-Brown Twiss measurement at P_{RF} = 4 dBm

Fig. S3 shows the Hanbury-Brown Twiss experiments of the T_A line when the acoustic field induced by the SAW is minimum. The period of the pulses is 2.019 ns, which is half of the SAW period since the modulated T_A line falls in the band pass of the monochromator twice. The value $g^2(0)$ = 0.066 is larger than the one shown in the main text but can be improved with longer integration time.

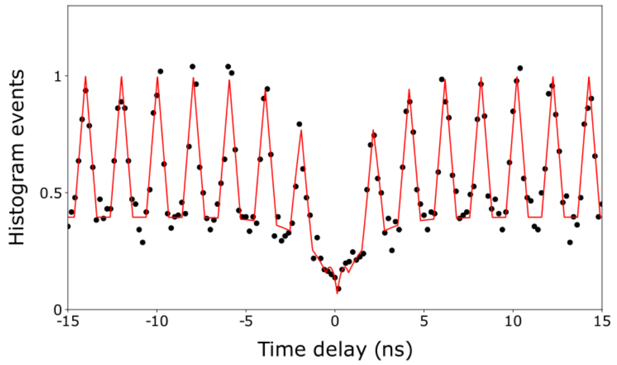

Fig. S3: Hanbury-Brown Twiss experiments of the T_A line when the SAW is generated at P_{RF} = 4 dBm and the monochromator set to 846.2 nm. The black dots are experimental values and the red curve is the fit based on the same function as described in the main text.

4. Insertion loss before and after SiO_2 deposition

To ensure that the IDT performance was not altered after removing the SiO_2 covering it, the insertion loss |S21| was compared before and after SiO_2 removal (Fig. S4). The resonance peak at 249 MHz has the same linewidth and amplitude, showing that the device performance has not been affected by the process in this region. The background outside the resonance is higher by approximately 5 dB after oxide removal, but this change does not affect the device operation. We note that the resonances found optically within the main lobe due to the acoustic cavity formed by the delay line are surprisingly not present in this electrical measurement.

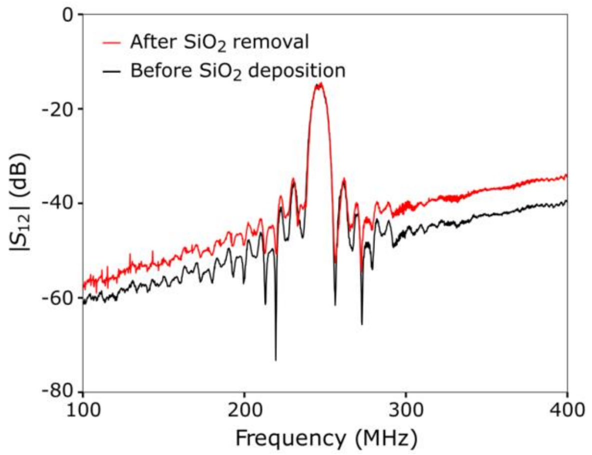

Fig. S4: Insertion loss of the delay line before oxide deposition (black) and after oxide removal on the IDT (red). The spectra were acquired when the sample was mounted in the cryostat and cooled to 1.6 K.

5. Strain profile for thick encapsulation layer

We simulated the displacements generated by the SAW on a 128° Y-X cut LN using a finite element method (COMSOL). The strain is then computed as

$$\varepsilon = \frac{\partial u_y}{\partial y} + \frac{\partial u_x}{\partial x},$$

where u_x and u_y are the displacements along the *x*-axis of the crystal and the direction normal to the surface, respectively.

Without encapsulation, the SAW is localized at the surface of the bulk/InP stack (Fig. S5(a)). Adding SiO_2 on the surface disrupts this localization as the wave also propagates in the encapsulation layer. This effect can be well visualized for thick encapsulation layer as shown in Fig. S5(b) for a 2 um deposition. As the wave propagates at the surface of the SiO_2, the strain in the now burried InP layer decreases.

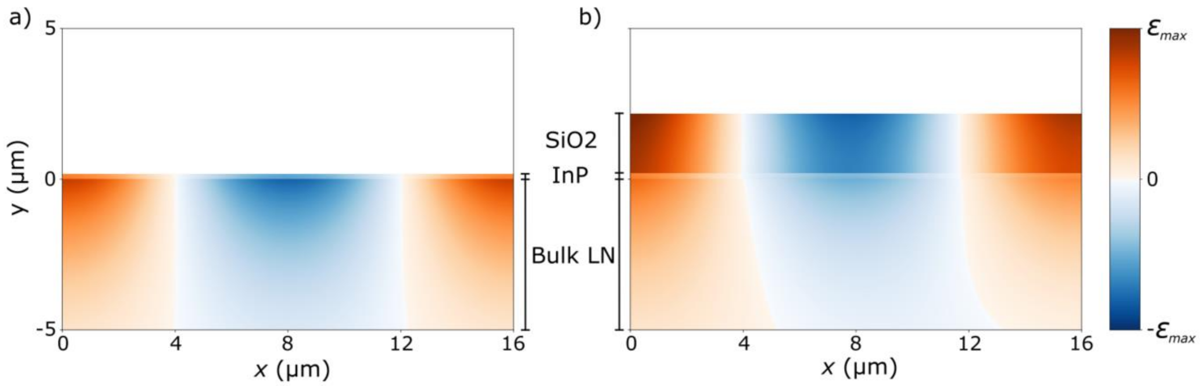

Fig. S5: Strain generated by the SAW (period 16 um) on a bare 128° Y-X cut LN substrate/200nm InP stack (a) and with a 2 um SiO_2 encapsulation layer (b).

6. Simulations of the nanowire-waveguide optical coupling

The coupling efficiency between the nanowire quantum dot and the LNOI waveguide was simulated with an eigenmode expansion solver (Lumerical). The geometry of the model is shown in Fig. S6(a) and the LNOI thickness, waveguide height and width were swept. The input port (port 1) was set to the fundamental TE mode of the nanowire and the output port (port 2) was the fundamental TE mode of

the LN waveguide. All the materials were considered loss-less. Better coupling is obtained for wider ridge with a thinner base and a smaller height (Fig. S6(b)).

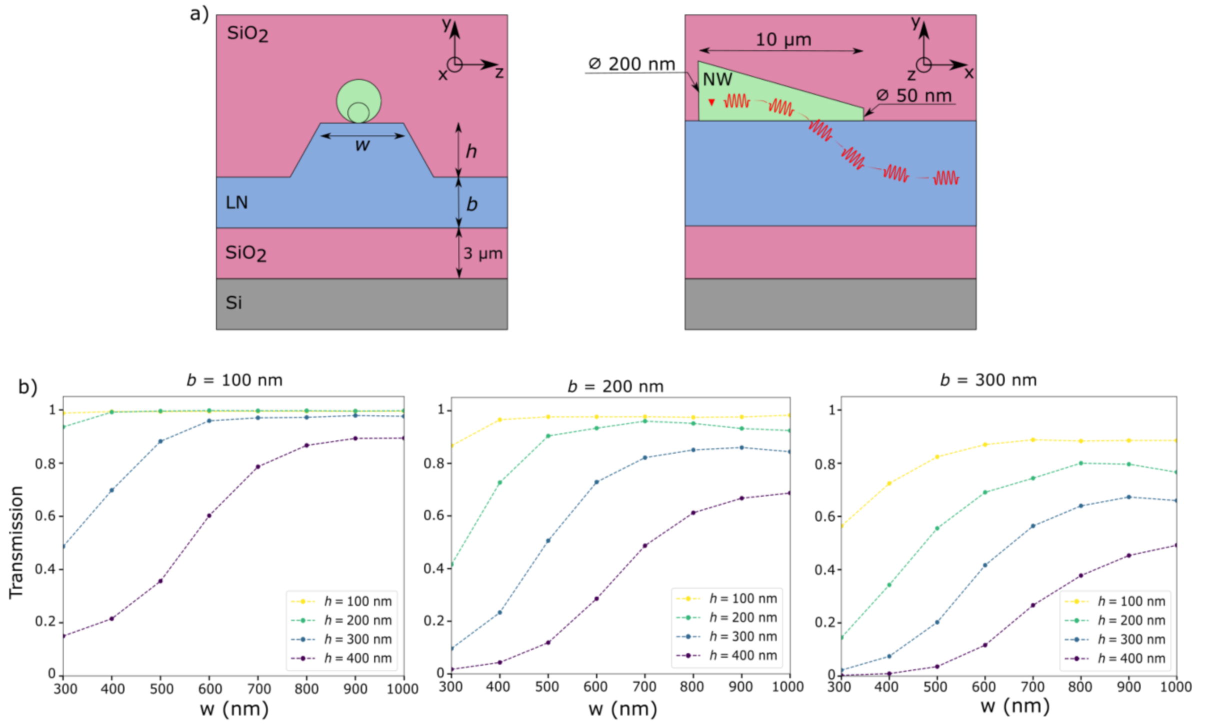

Fig. S6: Parametric study of the LNOI waveguide geometry. a) YZ view and YX view of the geometry and layer stack. The sidewall angle of ridge is fixed to 60°. b) The transmission, representing the coupling efficiency between the nanowire and the waveguide modes, is computed at 850 nm as a function of the waveguide width *w*, waveguide height *h*, and base thickness *b*.

7. Additional strain profiles for the LNOI architecture

The strain is computed as

$$\varepsilon = \frac{\partial u_y}{\partial y} + \frac{\partial u_z}{\partial z},$$

where u_y and u_z are the displacements along the *y*-axis and z-axis of the Y-cut LN crystal, respectively. The influence of the orientation of the nanowire QD with respect to the SAW propagation direction was investigated with the two extreme cases, parallel and perpendicular. We considered the same Y-cut LNOI as described in the main text, as well as the same nanowire geometry as shown in Fig. S6(a), with a 320 nm SiO_2 encapsulation (b = 200 nm, h = 200 nm, w = 800 nm). For both orientations, the strain field generated by the SAW couples to the nanowire QD when it is placed on the surface of a 200 nm thick LNOI (Fig. S7(a) and (b)). The strain at the center of the nanowire in the perpendicular case is 10 % larger than in the parallel case. Therefore, the nanowire orientation does not seem to have a significant impact on the final acousto-optical modulation.
To quantify the influence of the sidewall angle of the ridge waveguide on the strain field, we considered the worst-case scenario where the waveguide has straight walls (Fig. S7(c)). The strain field amplitude at the center of the nanowire is 98 % to that of the case with 60° sidewall angle.

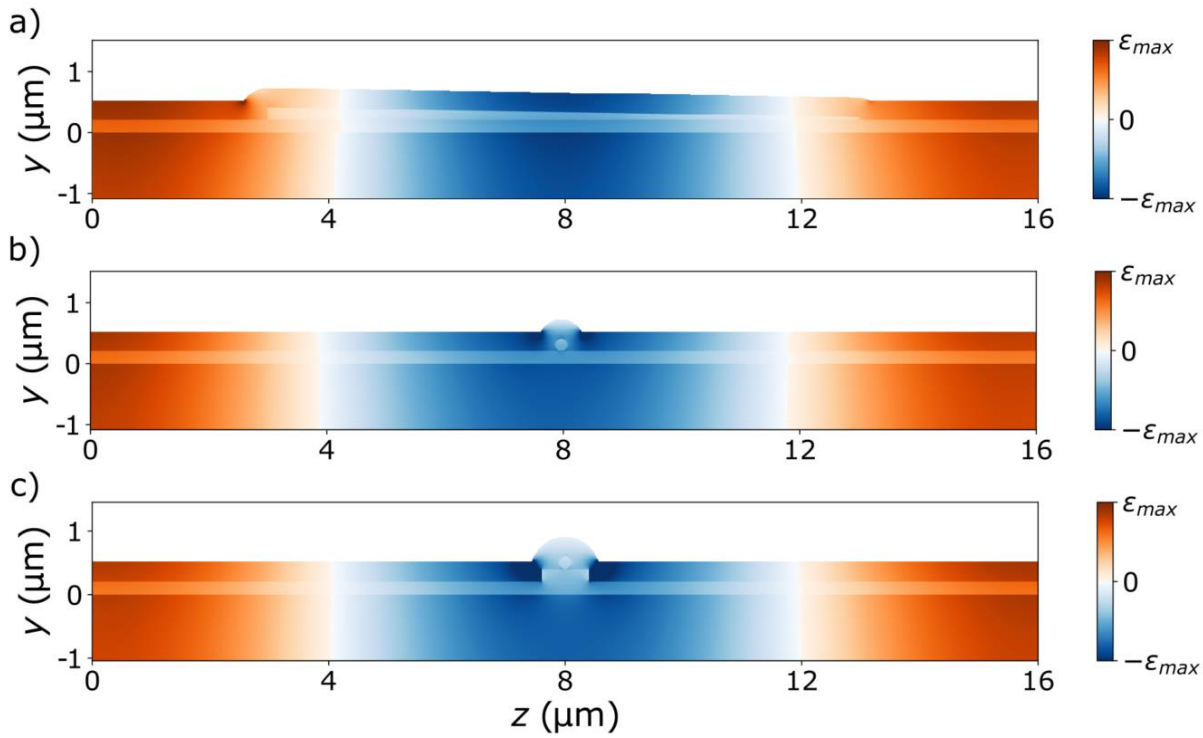

Fig. S7: Strain induced by the SAW launched along the *z*-axis of the LNOI cladded with 320 nm of SiO_2 and driven at 271.8 MHz (period 16 um) in three different configurations. a) The nanowire QD is on the 200 nm LNOI and parallel to the *z*-axis. b) The nanowire QD is on the 200 nm LNOI and perpendicular to the *z*-axis. c) The nanowire QD is on a LNOI waveguide with straight sidewalls and perpendicular to the *z*-axis. The scale bar of the strain is the same for all three subfigures. The axes correspond to those of the Y-cut LNOI.

As discussed in the main text, the encapsulation dome of SiO_2 on top of the waveguide (Fig. S8(b)) due to the conformal deposition decreases the strain inside the NW. Thicker encapsulation layers can alleviate this effect because the region of local minimum strain in the dome is further above the nanowire. It is mentioned in the main text that for a 320 nm encapsulation layer, the loss of strain at the center of the nanowire on top of the waveguide compared to the case where the nanowire is on the bare 200 nm thick LNOI is 43%. This loss decreases to 28% for 520 nm (Fig S8(b)) and 17% for 720 nm (Fig S8(c)) encapsulation. This thicker encapsulation remains in the range where the leakage of the SAW to the SiO2 encapsulation is negligible. In the extreme case where the dome is not considered as in Fig. S8(d), the loss of strain in the nanowire becomes minor.

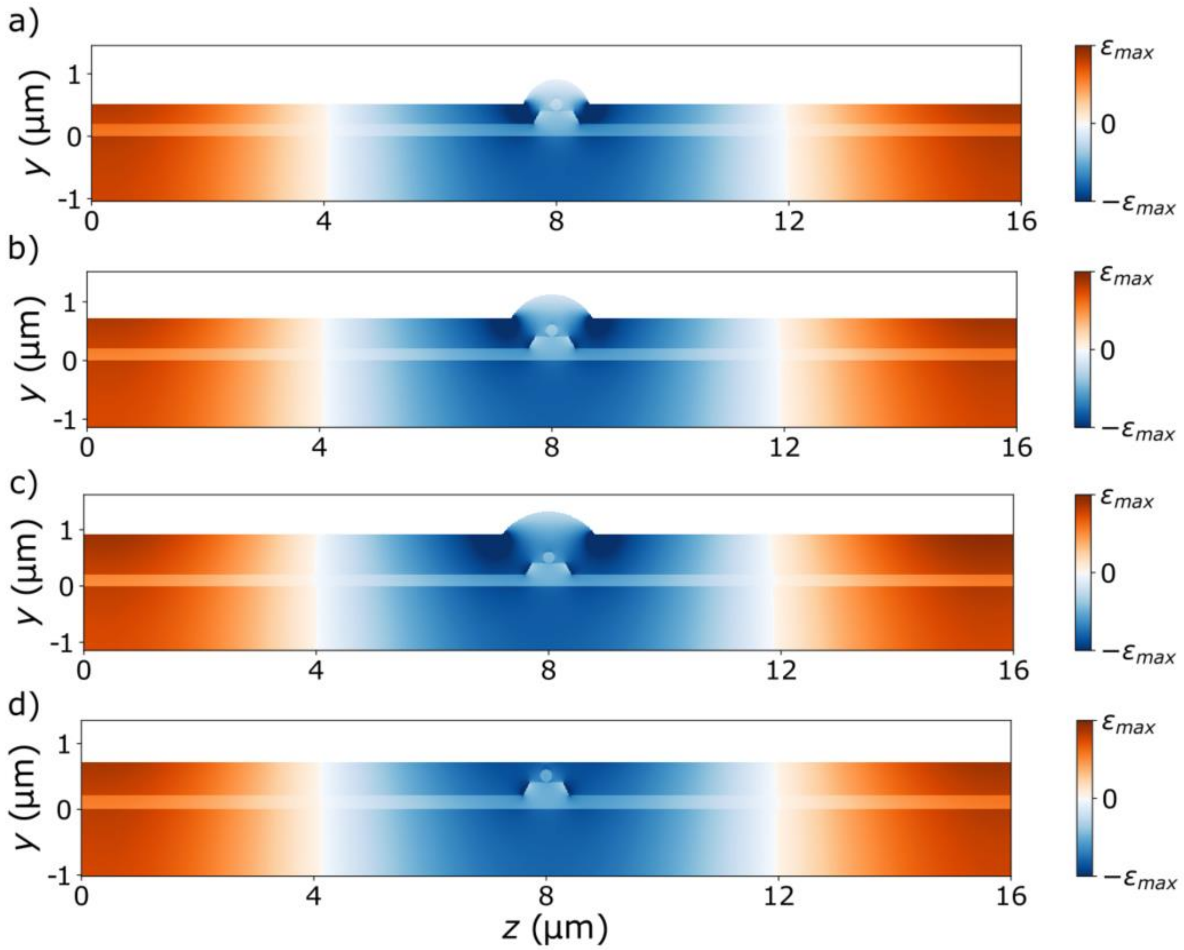

Fig. S8: Strain induced by the SAW (period 16 um) for 320 nm (a), 520 nm (b) and 720 (nm) SiO_2 encapsulating layer. The crystal axes correspond to those of the Y-cut LNOI. In d), the dome resulting from the conformal deposition has not been modelled as an extreme case. The scale bar of the strain is the same for all four subfigures.

8. Linewidth of the T_A peak before and after SiO_2 deposition

The linewidth measured with the spectrometer did not show noticeable change after SiO_2 encapsulation, as shown in Fig. S9.

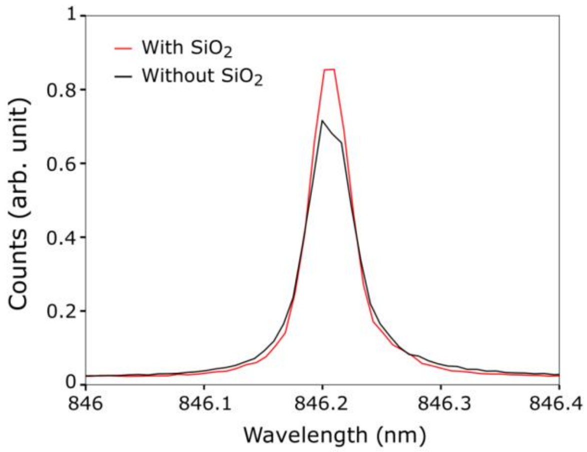

Fig. S9: Spectral linewidth of the T_A line before (black) and after (red) oxide deposition. The red curve has been shifted by 1.92 nm to compensate for the constant blueshift introduced by the oxide. No

significant broadening could be observed. The quantum dot was excited continuously with a HeNe laser at 150 nW.

9. Linewidth of the T_A peak before and after SiO_2 deposition

The lifetime of the T_A line was measured with pulsed HeNe laser at 5 MHz at 150 nW. Fig. S10 shows the experimental data together with a fit consisting of an exponential convoluted with the instrument response function (gaussian with a standard deviation of 244 ps). The extracted lifetime is 6.52 ± 0.1 ns.

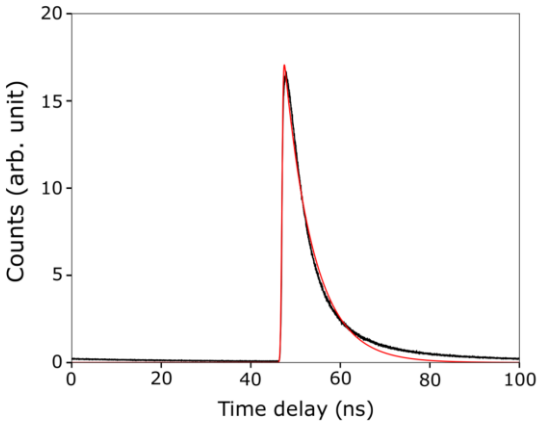

Fig. S10: Lifetime measurement (black line) of the T_A line and fitting function (red).

10. Strain modulation at 735 MHz

The spectral shift induced by the SAW generated at the third harmonic 735 MHz of the IDT is shown in Fig. S11.

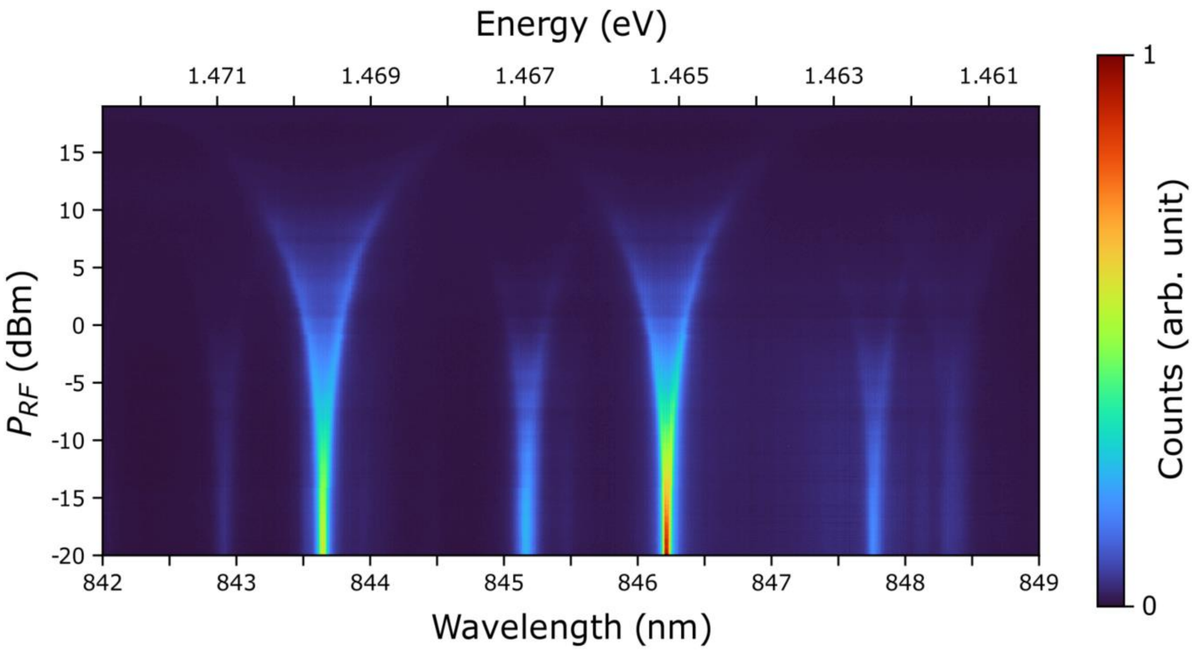

Fig. S11: Strain-induced energy broadening of the emission lines of the nanowire quantum dot by driving the IDT at the third harmonic 735 MHz. The nanowire QD shows a response similar to the modulation with the fundamental mode. The quantum dot was excited with a HeNe laser at 150 nW.

11. Estimation of the maximum number of single emitters tuned with SAW on the same chip

In this study, the emitter was placed in a delay line formed by two IDTs. Ultimately, only one IDT per emitter is enough to drive the SAW for independent tuning. If we consider the Y-cut, the IDTs should be placed next to one another along the X direction to generate SAWs along the Z direction. Two IDTs should not face each other in the Z direction to avoid overlap of the two generated SAWs. If the same acoustic aperture as in the text (180 um) is kept and each IDT is separated from its neighbors by 70 microns, 4 emitters can be integrated per mm. By reducing the acoustic aperture to diminish the footprint of the IDT while preserving its performance, the number of emitters could be pushed to 5 per mm.

References:

[1] Weiß, M.; Schülein, F. J. R.; Kinzel, J. B.; Heigl, M.; Rudolph, D.; Bichler, M.; Abstreiter, G.; Finley, J. J.; Wixforth, A.; Koblmüller, G.; Krenner, H. J. Radio Frequency Occupancy State Control of a Single Nanowire Quantum Dot. Journal of Physics D: Applied Physics, 2014, 47, 394011.

[2] Schmidt, H.; Kunze, R.; Weihnacht, M.; Menzel, S. Investigation of acoustomigration effects in Al-based metallizations. Proceedings of the IEEE Ultrasonics Symposium 2002, 1, 415–418.

[3] Paquit, M.; Djoumi, L.; Vanotti, M.; Soumann, V.; Martin, G.; Blondeau-Patissier, V.; Baron, T. Displacement of Microparticles on Surface Acoustic Wave Delay Line Using High RF Power. IEEE International Ultrasonics Symposium, IUS 2018, 2018-Octob.

[4] Bühler, D. D.; Weiß, M.; Crespo-Poveda, A.; Nysten, E. D.; Finley, J. J.; M ̈uller, K.; Santos, P. V.; de Lima, M. M.; Krenner, H. J. On-chip generation and dynamic piezo-optomechanical rotation of single photons. Nature Communications 2022, 13, 1–11.
